# Large-Scale Profiling on lncRNAs in Human Platelets: Correlation with Platelet Reactivity

**DOI:** 10.3390/cells11142256

**Published:** 2022-07-21

**Authors:** Yeying Sun, Rongrong Liu, Xiangwen Xia, Luchuan Xing, Jing Jiang, Weihua Bian, Wendy Zhang, Chunhua Wang, Chunxiang Zhang

**Affiliations:** 1 US-China Institute for Translational Medicine, College of Pharmacy, Binzhou Medical University, Yantai 264000, China; sunyy21cn@163.com (Y.S.); liurongrong371423@163.com (R.L.); xiawen858@hotmail.com (X.X.); 15275531216@163.com (L.X.); jingjiang1974@sina.com (J.J.); bian_1005@163.com (W.B.); chunhuawang508@126.com (C.W.); 2 Department of Biomedical Engineering, School of Medicine, The University of Alabama at Birmingham, Birmingham, AL 35233, USA; wendyzhang016@gmail.com; 3 Department of Cardiology, The Affiliated Hospital of Southwest Medical University, Key Laboratory of Medical Electrophysiology of Ministry of Education, Institute of Cardiovascular Research, Nucleic Acid Medicine of Luzhou Key Laboratory, Metabolic Vascular Disease Key Laboratory of Sichuan Province, Southwest Medical University, Luzhou 646000, China

**Keywords:** platelets, long noncoding RNAs, hyperreactive, hyporeactive, platelet function

## Abstract

Recently, long noncoding RNAs (lncRNAs) have been key regulators for both mRNAs and proteins in nucleated cells. However, the expression profiles of lncRNAs in non-nucleated cells such as platelets are currently unclear. In this study, we determined the expression profiles of lncRNAs in human platelets. We found that 6109 lncRNAs were expressed in human platelets. Interestingly, 338 lncRNAs were differentially expressed in hyperreactive and hyporeactive platelets. Bioinformatics’ analysis revealed that these aberrantly expressed lncRNAs might be related to platelet activity and other platelet functions. To provide a proof of concept, we measured the expression levels of PARLncRNA-1, a down-regulated lncRNA of hyperreactive platelets, in platelets from 12 patients with acute myocardial infarction and their controls. We found that the lncRNA was also significantly down-regulated in platelets from patients, which was partially reversed by treatment with aspirin a known antiplatelet drug. LncRNAs may represent a novel class of modulators for platelet functions.

## 1. Introduction

Long noncoding RNAs (lncRNAs) are defined as non-coding RNAs longer than ~200 nucleotides and lack protein-encoding capacity [1,2]. Based on their location and function, LncRNAs are regularly classified into five subclasses, which include intergenic, intronic, sense overlapping, anti-sense, and bidirectional lncRNAs [1,2,3,4]. The increasing evidence has clearly shown that lncRNAs have strong biological functions and may be involved in all biological and disease processes [5,6].

Platelets are blood cells that play critical roles in thrombosis, inflammation, hemostasis, atherogenesis, tissue injury repair, antimicrobial host defense, and tumor growth and metastasis [7,8]. As there are no nuclear DNAs, epigenetic regulation may play more important roles in platelet functions to maintain a proteome and adapt to environmental situations over their lifespan. In this respect, platelets retain many of the RNA metabolic processes through posttranscriptional mechanisms. It is clear that in the absence of transcription, the dependence on post-transcriptional mechanisms to regulate gene expression via non-coding RNAs may make up a greater proportion of the platelet transcriptome than observed in cells with nuclei. Indeed, many small non-coding RNAs such as microRNAs are found in platelets and are strong regulators for platelet functions [9]. Recently, it was found that a large number of lncRNAs are present in platelets by high-throughput sequencing technology [10]. However, little is known about the function of lncRNAs in platelets.

As lncRNAs could be located both in nuclear and cytoplasm in a cell, which could maintain and regulate mRNAs and protein levels by multiple and diverse mechanisms, we thus hypothesize that human platelets may contain a significant amount of lncRNAs which are associated with their functions. More and more evidence showed that lncRNAs play an important role in platelet reactivity. ENST00000433442 was considered as significantly correlated with high platelet reactivity [11]. Knockdown of lncRNA metallothionein 1 pseudogene 3 (MT1P3) can inhibit platelet activation and aggregation in an animal model of diabetes by reducing P2Y12 expression [12].

In the current study, we identified via genome-wide large-scale next-generation deep sequencing, that platelets have a large amount of lncRNAs. To provide the potential functional links between platelet lncRNAs and platelet functions, we compared the expression profiles of lncRNAs between hyperreactive and hyporeactive platelets in the general population. Gene ontology (GO), Kyoto Encyclopedia of Genes and Genomes (KEGG) pathway analysis, lncRNA-mRNA co-expression network construction, and novel lncRNAs target gene prediction were performed for their links with other genes and signaling pathways associated with platelet functions. Finally, to provide a proof of concept under disease conditions, we compared the expression levels of a lncRNA in platelets from patients with acute myocardial infarction, which is a thrombosis-inflammation-related disease, and with those from their age- and sex-matched controls.

## 2. Materials and Methods

### 2.1. Healthy Participants, Patients and Blood Samples

Eighty-six healthy subjects were recruited through local print advertisements at Binzhou medical university. Participants would be excluded if they were taking medication (e.g., anti-histamines, aspirin, non-steroidal anti-inflammatory medication) known to interfere with platelet function two weeks before the blood draw. Subjects were asked to fast overnight and to refrain from intensive exercise before early-morning phlebotomy. After resting comfortably for at least 10 min, subjects were phlebotomized. Blood was collected through a 19-gauge needle into a vacutainer tube containing 3.8% sodium citrate after the first 2 mL was discarded. The participants were categorized as having hyperreactive (*n* = 46) or hyporeactive (*n* = 40) platelets if the maximal aggregation in response to epinephrine (1.5 μM), collagen (20 μg/mL), and adenosine diphosphate (ADP) (10 μM) was >60% or <40%, respectively, as described [13,14].

In the second set of studies, 12 patients with acute myocardial infarction (AMI) and 12 age- and sex-matched healthy control participants were enrolled. In patients, the blood samples were obtained before and after 3 days of treatment with the anti-platelet drug aspirin. The AMI inclusion criteria were ischemic chest pain lasting >30 min, positive biochemical markers (cardiac troponin I [cTnI] > 0.1 ng/mL), presentation within 12 h after the onset of symptoms, electrocardiogram showing a new ST-segment elevation of 0.1 mV in at least two contiguous leads. All AMI patients were confirmed by angiography and underwent primary percutaneous coronary intervention (PCI). 

This study was approved by the institutional review committee of the Binzhou medical university, and the participants gave informed consent, which conformed with the Declaration of Helsinki.

### 2.2. Platelet Aggregation Assay

Platelet aggregometry was determined based on the method as described [14]. Briefly, blood was centrifuged for 15 min at 150 g to obtain platelet-rich plasma (PRP). Platelet-poor plasma (PPP) was obtained at 400 g for 5 min. The number of platelets in the PRP was counted using a hemocytometer and adjusted to 200,000 and 250,000 platelets/μL using PPP. Platelet aggregation was measured with a Chrono-Log aggregometer (Chrono-Log, Havertown, PA, USA), by using epinephrine (1.5 μM), collagen (20 μg/mL), and adenosine diphosphate (ADP) (10 μM) as stimulators. Briefly, the samples were incubated for 3 min at 37 °C. After agonists were added, the reaction lasted 10 min. Maximal aggregation (%) and the slope of the aggregation curve were collected.

### 2.3. Platelet Isolation, Purification and RNA Extraction

Platelets were isolated and purified via density centrifugation and immune depletion of CD45^+^ cells using human CD45 MicroBeads reagent (Miltenyi Biotec GmbH, Bergisch Gladbach, Germany) according to manufacturer’s instructions. [15,16]. At last, a MACS^®^ bead separation system (Miltenyi Biotec GmbH) was used to collect leukocyte-depleted platelets with purity >99.99%. Platelet RNA was extracted RNA with AG RNAex pro (Accurate Biotechnology (Hunan) Co., Ltd., Changsha, China). An Agilent Bioanalyzer was used for RNA quality control.

### 2.4. Genome-Wide Large-Scale Next-Generation Deep Sequencing of lncRNAs and mRNAs

To determine the expression profiles of lncRNAs and mRNAs in platelets, RNAs from both hyperreactive and hyporeactive platelets were used. Three micrograms of RNA per sample were used as the input material for the RNA sample preparations after ribosomal RNA (rRNA) depletion and DNase I treatment. The expression profiles of lncRNAs and mRNAs were determined via genome-wide large-scale next-generation deep sequencing by using the Illumina platforms according to the Illumina Solexa transcriptome sequencing protocol. The libraries were sequenced at the Anoroad Genome (Beijing, China) on an Illumina Hiseq 2000 platform, and 90-bp paired-end reads were generated. lncRNAs and mRNAs with a log2Ratio ≥ 1, a *p* < 0.05, and a false discovery rate (FDR) < 0.1 were identified as significantly differentially expressed. Sequencing data were mapped against the NONCODE database of lncRNAs, using default parameters and the web-based tool miRMaster. LncRNAs were further classified into two categories: known lncRNAs and novel lncRNAs. According to the database, the known lncRNAs were identified as different categories such as lincRNA and antisense. *p*-values were generated by the Kruskal–Wallis test and used for evaluation of gene expression changes after Benjamini-Hochberg adjustment for multiple testing. To identify the potential novel lincRNAs, the assembled transcripts that overlapped with known mRNAs and lncRNAs (NONCODE v5.0) were removed using the tool Cuffcompare. Any new transcripts with a length ≤ 200 nucleotides were removed, and the coding potential of the selected transcripts was evaluated using the Coding-Potential Assessment Tool (CPAT) method. Those transcripts with CPAT scores ≥ 0.3 were discarded.

### 2.5. Quality Control and RNA-Seq Data Processing

To assess the quality of RNA-seq data, each base in the reads was assigned a quality score (Q) by Fast QC with a Phred-like algorithm. Clean data were obtained by removing reads containing adapters, low-quality reads (>15% of bases whose Q scores were <19), rRNA mapping reads and reads containing over 5% poly-N from the raw data. The clean data were mapped to the human genome GRCh38 (hg38) by TopHat2 (v2.0.13) with default parameters. The transcripts were assembled using Cufflinks (v2.2.0) according to the instructions provided. The sequencing data discussed in this publication have been deposited in NCBI’s Gene Expression Omnibus [17] and are accessible through GEO Series accession number GSE97348. All the identified lncRNAs in hyperreactive and hyporeactive platelets were listed in the deposited online data (https://www.ncbi.nlm.nih.gov/geo/query/acc.cgi?acc=GSE97348, accessed on 2 April 2020).

### 2.6. Gene Ontology (GO) and Encyclopedia of Genes and Genomes (KEGG) Pathway Analysis

Gene ontology was used to identify the biological implications of significant or representative differentially expressed genes. Those genes with significant differential expression in hyperreactive and hyporeactive platelets were used for GO analysis (www.geneontology.org, accessed on 1 June 2020). The ontology covers three domains: biological process, cellular component, and molecular function. Fisher’s exact test was used to determine whether the overlap between the differential expression list and the GO annotation list was greater than would be expected by chance. Pathway analysis of lncRNA co-expressed mRNA is a functional analysis that maps genes to KEGG pathways (www.genome.jp/kegg, accessed on 1 June 2020). The *p*-value (EASE-score, Fisher’s exact test *p*-value, or hypergeometric *p*-value) denotes the significance of the GO term and thus the pathway that correlates with the conditions. The lower the *p*-value, the more significant the pathway and GO term. A *p*-value ≤ 0.05 is recommended.

### 2.7. LncRNAs Target Gene Prediction

Differentially expressed novel lncRNAs were selected for cis- and trans-target gene prediction and were analyzed with the GO and KEGG enrichment of targets. The mRNAs were identified as “cis-regulated target genes” when the mRNA loci were within a 20-kb window up or downstream of the given lncRNA. It should be noted that it was not a precise window up or downstream of the given lncRNA. Previous studies used different windows such as 100-kb, 50 kb and 10 kb, etc. [18,19,20]. Therefore, we selected a 20-kb window which is among the established ranges. For the trans-acting analysis, the BLAST software was used to identify possible target genes for lncRNAs. RNAplex program was performed to quickly localize possible hybridization sites for a query RNA in large RNA databases by applying a simpler energy model in the first screening phase followed by a full energy model to refine potential binding sites.

### 2.8. Quantitative Reverse Transcription-Quantitative Polymerase Chain Reaction (qRT-qPCR) of Selected lncRNAs

Total RNAs from platelets were reverse transcribed to cDNA using Evo M-MLV RT Kit (Accurate Biotechnology (Hunan) Co., Ltd., Changsha, China). qRT-PCR for selected LncRNAs were performed on cDNAs using SYBR Green Pro Taq HS (Accurate Biotechnology (Hunan) Co., Ltd., Changsha, China). GAPDH was used as the internal control. The relative gene expression of lncRNAs was calculated by comparing the threshold cycle (C_T_). Each sample was tested in triplicate.

### 2.9. Statistics

All the data were presented as mean ± standard deviation (for deep sequencing data) or standard error (for qRT-PCR data). For relative gene expression, the mean value of the vehicle control group is defined as 100%. Two-tailed unpaired Student *t*-tests and ANOVAs were used for statistical evaluation of the data. SPSS 19.0 was used for data analysis. A *p* < 0.05 was considered significant.

## 3. Results

### 3.1. lncRNAs Are Highly Expressed in Human Platelets

To identify hyperreactive and hyporeactive groups, 86 healthy subjects were recruited for aggregometry analysis. The subjects were considered to have a hyperreactive platelet phenotype if over 60% aggregation was observed using epinephrine (1.5 μM), collagen (20 μg/mL), and ADP (10 μM) as agonists. On the contrary, individuals were categorized as having a hyporeactive platelet phenotype if the aggregation induced by each agonist was less than 40%. From the results of aggregometry analysis, three hyperreactive subjects and three hyporeactive subjects were selected for the profiling of platelet lncRNAs profiling. Table S1 shows the differential platelet aggregation response to agonists for the six selected individuals. The RNA extraction from the three hyperreactive subjects was mixed in the same amount to consist of the hyperreactive pool. The other hyporeactive pool came from the three hyporeactive ones in the same way. We used high-throughput sequencing technology to identify the lncRNAs in platelets and the differential platelet lncRNAs that were differentially expressed in hyperreactive and hyporeactive platelet phenotypes.

**Figure 1:**
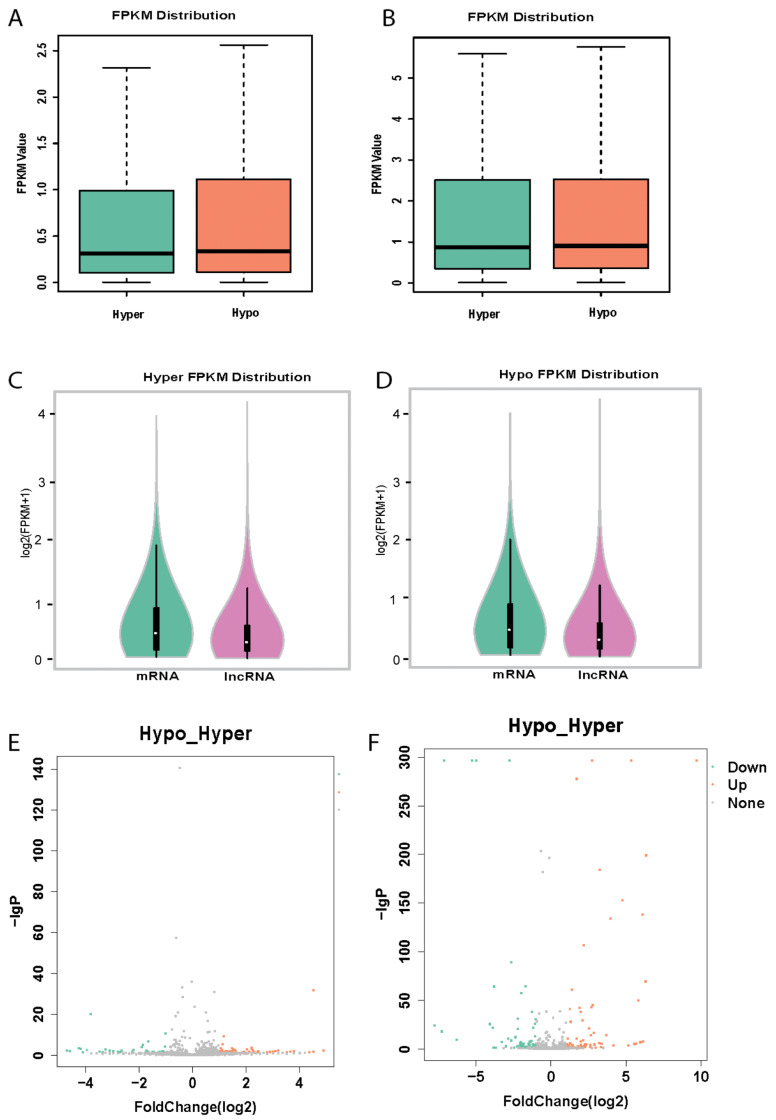
LncRNAs and their expression changes in platelets determined by the RNA-seq data set. (**A**) Box plot showing the distribution of the FPKM (fragments per kilobase of exon per million fragments mapped) values of known lncRNAs. (**B**) Box plot showing the distribution of the FPKM of novel lncRNAs. (**C**) Compare the mRNA and lncRNAs distribution in hyperreactive platelets. (**D**) Compare the mRNA and lncRNAs distribution in hyporeactive platelets. (**E**) Volcano plot of differentially expressed known lncRNAs between hyperreactive and hyporeactive platelets Down or up mean lower or overexpression in hyporeactive platelets compared to hyperreactive platelets (Hypo vs. Hyper). (**F**) Volcano plot of differentially expressed novel lncRNAs (Hypo vs. Hyper). Red dots represent up expressed RNAs and green dots represent down expressed RNAs with at least two-fold change and corrected *p*-value <0.05. Orange and cyan dots indicate significantly upregulated and downregulated lncRNAs, respectively.

The results of deep sequencing identified that lncRNAs were highly expressed in human platelets. In total, 6109 lncRNAs were found in both hyperreactive and hyporeactive platelets, among them, 4942 were known lncRNAs and 1167 were novel lncRNAs (not annotated previously in the NOCODE database). The top 30 most abundant lncRNAs were listed in Table 1. The distribution of the amount of lncRNAs as shown by Fragments per Kilobase Millon Mapped Fragments (FPKM) in both hyperreactive and hyporeactive platelets was demonstrated in Figure 1A,B, which was comparable with that of mRNAs in platelets (Figure 1C,D).

### 3.2. Platelet lncRNAs Are Differentially Expressed in Hyperreactive and Hyporeactive Platelets

To determine the potential links between the expression of lncRNAs and the function of platelets, the expression profiles of hyperreactive and hyporeactive platelets were compared. Interestingly, deep sequencing revealed differential expression of lncRNAs between hyperreactive and hyporeactive platelets. In total, 338 lncRNAs differentially expressed between hyperreactive and hyporeactive platelets. Among them, the expression, and fold changes of 193 differentially expressed known lncRNAs (Hypo vs. Hyper) were listed in Supplementary Table S1 and in their volcano plot (Figure 1E), whereas, the expression, and fold changes of 145 novel platelet lncRNAs (Hypo vs. Hyper) were listed in Supplementary Table S2 and a volcano plot (Figure 1F).

### 3.3. Gene Ontology Enrichment Analysis of Differentially Expressed Platelets mRNAs

**Figure 2:**
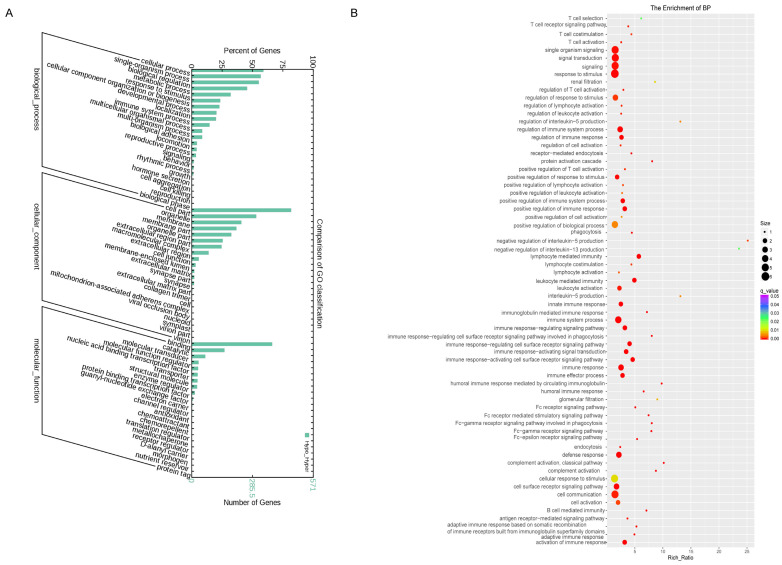
Gene ontology enrichment analysis of differentially expressed platelets mRNAs. (**A**) GO analysis of differentially expressed mRNAs in hyperreactive and hyporeactive platelets. (**B**) GO enrichment score graph of differentially expressed mRNAs in hyperreactive and hyporeactive platelets as regards biological processes.

Studies have shown that lncRNAs could regulate the expressions of neighboring and overlapping coding genes. Therefore, GO analysis was performed to investigate the biological processes, cellular components, and specific molecular functions of all differentially expressed mRNAs as shown by the bar graph (Figure 2A). GO enrichment analysis of significantly differentially expressed mRNAs in hyperreactive and hyporeactive platelets could reveal the probable role or function of related dysregulated lncRNAs. The data of GO enrichment analysis showed that multiple genes were involved, especially the genes of antigen binding, receptor activity, signaling receptor activity, transmembrane signaling receptor activity, MHC class II receptor activity, signal transducer activity, molecular transducer activity, estrogen receptor activity, G-protein coupled receptor activity, and ATP-dependent DNA helicase activity, which are associated with platelet activity and other functions [21,22,23,24] as shown in the GO enrichment score graph (Figure 2B).

### 3.4. KEGG Target Pathway of the Differentially Expressed Platelets lncRNAs

**Figure 3:**
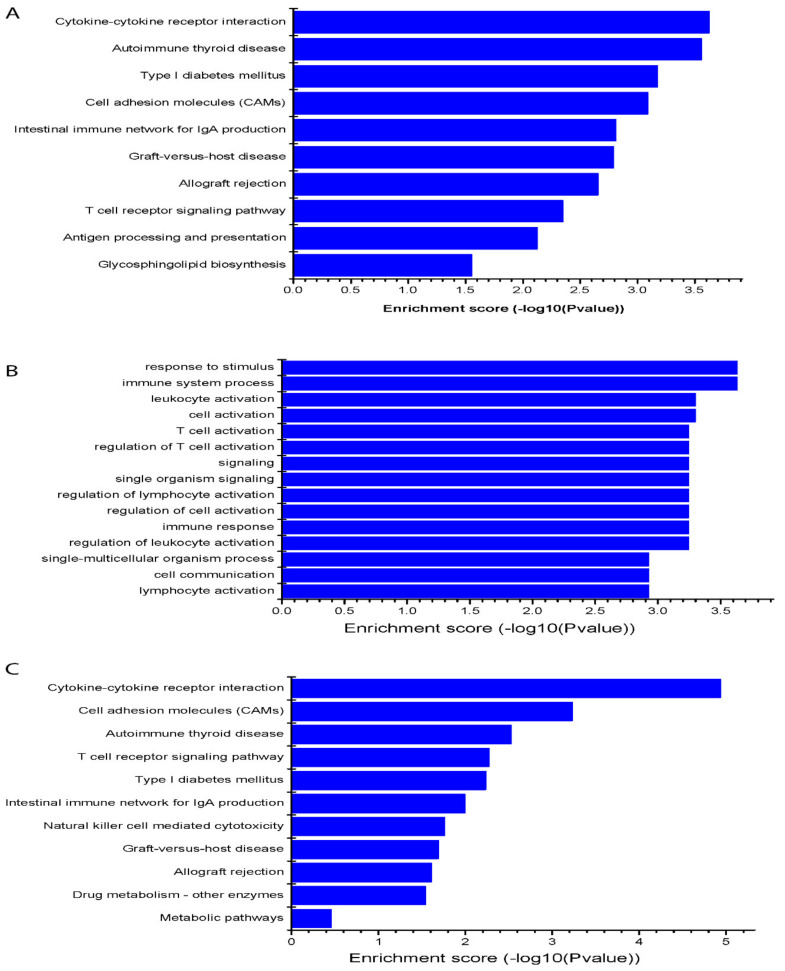
KEGG pathway analysis of target pathways of differentially expressed platelets lncRNAs and target gene prediction of a novel lncRNA. (**A**) The most enriched pathway among up-regulated and down-regulated mRNAs. (**B**) GO enrichment score graph of target genes of lnc_1285 as regards biological processes. (**C**) KEGG pathway analysis of target genes of lnc_1285.

KEGG allowed us to determine through the biological pathways that there is a significant enrichment of differential expressed mRNAs related to studied lncRNAs. As shown in Figure 3A, some pathways were involved, in which cytokine–cytokine receptor interaction, cell adhesion molecules, autoimmune thyroid disease, type I diabetes mellitus, intestinal immune network for IgA production, graft-versus-host disease, allograft rejection, T cell receptor signaling pathway, antigen processing and presentation, antigen processing and presentation, and glycosphingolipid biosynthesis were among the top score pathways. They are associated with platelet functions and platelet-mediated biological effects of the differentially expressed lncRNAs between hyperreactive and hyporeactive platelets [18,22,23] as shown by the KEGG enrichment score graph (Figure 3A).

### 3.5. LncRNA Regulated-mRNAs via Target Gene Prediction Are Involved in Platelets Aggregation and Other Functions

LncRNAs can regulate the expression of coding genes. As a result, the functions of these coding genes might provide insight into these differentially expressed lncRNAs. As an example, we predicted the target genes of one novel lncRNA, lnc_1285 by cis- and trans- analysis, and then analyzed the GO and KEGG enrichment of targets. We found that the most enriched GO terms associated with targeted mRNAs of the novel lncRNA were the response to stimulus, immune system process, leukocyte activation, cell activation, T cell activation, regulation of T cell activation, signaling, single organism signaling, regulation of lymphocyte activation, regulation of cell activation, immune response, which are related to platelet activity [18,19,20,21] as shown in the GO enrichment score graph (Figure 3B). KEGG pathway analysis was applied to gain a deep understanding between the targeted mRNAs and cell pathways of this novel lncRNA. As a result, the most enriched pathways were involved, including cytokine-cytokine receptor interaction, cell adhesion molecules, and T cell receptor signaling pathway, which are associated with platelet functions [21,25,26] as shown by the KEGG enrichment score graph (Figure 3C).

### 3.6. Validation of Differentially Expressed lncRNAs in Hyperreactive and Hyporeactive Platelets

**Figure 4:**
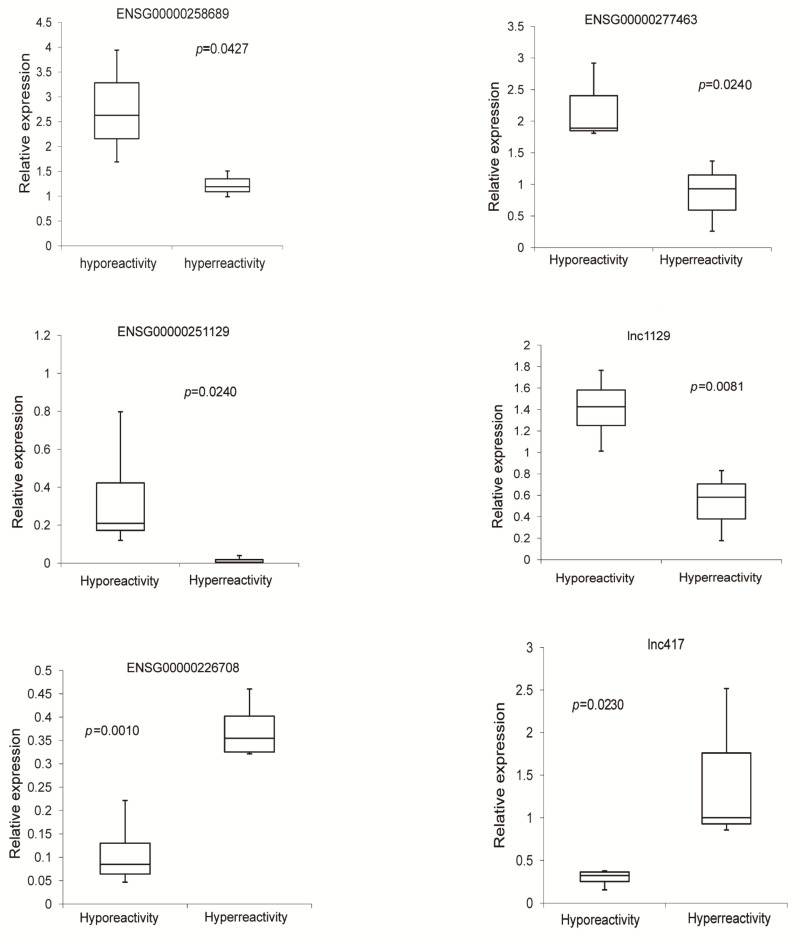
Quantitative real-time PCR validation of differentially expressed 6 lncRNAs.

To validate our results of deep sequencing, six abundant lncRNAs, which were differentially expressed lncRNAs in hyperreactive and hyporeactive platelets identified by deep sequencing analysis, were selected. Their expression levels were further determined by qRT-PCR. The primers used were listed in Supplementary Table S3. Results showed that the expression changes of these lncRNAs were consistent with those from deep sequencing analysis (Figure 4).

### 3.7. The Expression of ENSG00000258689, a Down-Regulated lncRNA in Hyperreactive Platelets Is Significantly Decreased in Platelets from Patients with AMI

**Figure 5:**
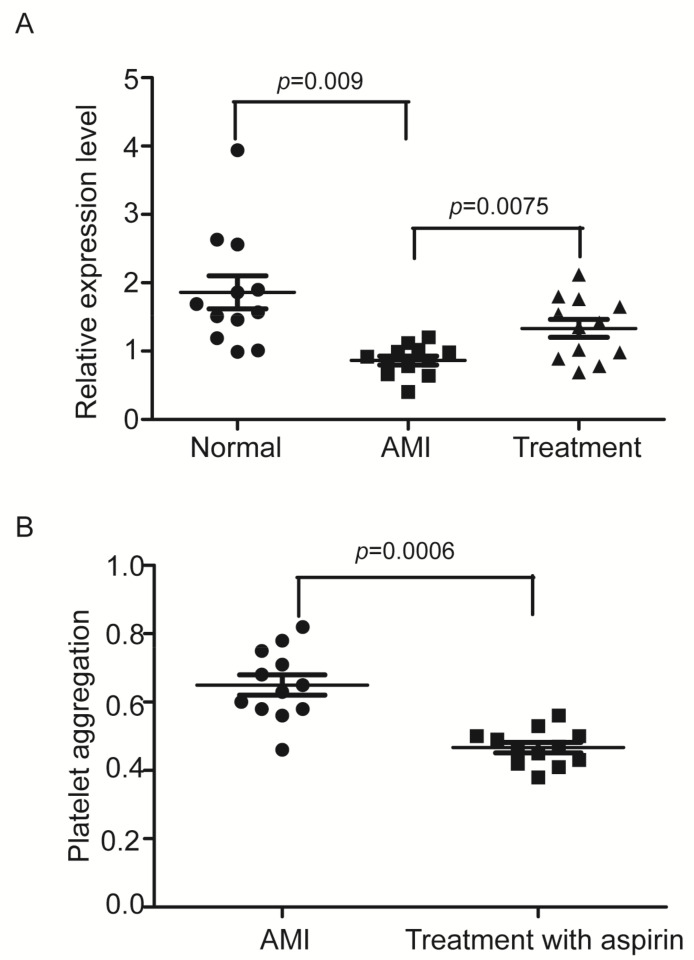
lncRNA, ENSG00000258689 levels in normal platelets and AMI platelets with the enhanced platelet aggregation. (**A**) ENSG00000258689 expression was decreased in AMI patients, which was partially reversed by treatment with aspirin (n = 12). (**B**) Comparison the platelet aggregation before and after treatment with aspirin (n = 12). (ADP) (10 μM) was used as an agonist.

ENSG00000258689 was the most abundant lncRNA among all the six detected lncRNAs via qRT-PCR in platelets and its expression was significantly downregulated in hyperreactive platelets. To provide a proof of concept, we thus measured the expression levels of ENSG00000258689, in platelets from 12 patients with AMI and their aged and sex-matched controls, we found that the LncRNA was also remarkably down-regulated in platelets from patients, which was partially reversed by treatment with aspirin a known antiplatelet drug as shown in Figure 5A. As expected, the platelet reactivity was increased in AMI patients, which was partially reversed by treatment with aspirin (Figure 5B). Clearly, ENSG00000258689 is a platelet-enriched lncRNA and its expression is associated with platelet activation. We thus named this lncRNA the platelet activation-related lncRNA 1 (PARLncRNA-1).

## 4. Discussion

In the present study, we investigated the genome-wide expression profile of platelet lncRNAs. We found a significant number and amount of lncRNAs in human platelets. It should be noted that the relative abundance of mRNAs and lncRNAs in platelets might be different from those in other cells due to the lack of nuclei. The genomic ids/names, expression levels, genomic locations, and categories of these platelet-expressed lncRNAs were described. It is well-established that the tissue/cell-specific expression and function is one important characteristic of lncRNAs. The expression signature of lncRNAs in human platelets described in this study could be a prerequisite to starting the new research field in platelet biology. Recently, Heililahong et al. demonstrated that 2923 lncRNAs were detected in the platelet concentrates (PC) during storage by high-throughput sequencing technology. Moreover, the levels of 42 species increased and the levels of 28 species decreased during storage from day 0 to day 4 [27]. The differential expression levels imply that lncRNAs maybe explore some biological functions in platelet storage damage.

Based on the activity, the platelets in the general population could be categorized into hyperreactive and hyporeactive platelets, whereas platelet hyperreactivity is an established risk for many human diseases such as stroke and heart diseases [28,29]. lncRNAs have been proved to be involved in platelet activity. lncRNA ENST00000433442 was demonstrated as significantly correlated with high platelet reactivity, which could be influenced by double antiplatelet therapy [11].

To provide the potential links between these platelet lncRNAs and platelet functions, we determined the difference in lncRNA expression profiles between hyperreactive and hyporeactive platelets. Excitingly, the expression signatures of lncRNAs in platelets with differential functions were much different. To further provide the links of platelet lncRNAs and their functions in platelets, we determined the genome-wide mRNAs in both hyperreactive and hyporeactive platelets and performed the detailed GO analysis and KEGG analysis of the potential associate/target genes and signaling pathways of differentially expressed platelets lncRNAs. These co-expression and bioinformatics analyses demonstrated that multiple genes/signaling pathways which are related to platelet functions were affected by these differentially expressed platelet lncRNAs [18,19,20,21,22]. In addition, some genes/pathways related to platelet-mediated effects on other cells/tissues were also affected by these differentially expressed platelet lncRNAs, which need to be studied in the future. To further provide the potential functional links of platelet lncRNAs and platelet coding genes. We analyzed the target genes of a novel platelet lncRNA, lnc_1285, by target gene prediction. We found that its target genes were indeed related to platelet functions.

Evidence has suggested that lncRNAs in circulating platelets could be a biomarker or therapeutic target in many diseases. Ye et al. reported four lncRNAs, LNCAROD, SNHG20, LINC00534, and TSPOAP-AS1, upregulated in platelets of colorectal cancer (CRC) patients. They might be developed biomarkers for CRC diagnostics [30]. To provide a direct link of platelet lncRNAs with cardiovascular disease, we determine the expression of ENSG00000258689, a down-regulated lncRNA in hyperreactive platelets, in patients with AMI. We found that the expression of ENSG00000258689 in platelets from AMI patients was also significantly down-regulated, compared with that in normal control subjects. Interestingly, the aberrant expression of ENSG00000258689 in AMI patients could be partially reversed by an antiplatelet drug, aspirin. The roles of ENSG00000258689 in platelet functions and cardiovascular diseases and the molecular mechanisms involved should be studied in the future. In the present study, our data was focused on the transcriptional and RNA levels. As lncRNAs could bind and affect the protein expression directly, protein studies should also be performed in future studies.

## 5. Conclusions

Platelets have no nuclear DNAs; however, platelets could effectively maintain their proteome and respond very well to many environmental situations over their 8- to 9-day lifespan. The anucleated platelets retain many of the RNA metabolic processes of nucleated cells and could regulate RNA levels through posttranscriptional mechanisms including microRNAs. In this study, we have identified, for the first time, that lncRNAs may represent a novel class of gene regulators in platelets. Indeed, a significant amount of lncRNAs are found in platelets. As no nuclear DNAs in the non-nucleated platelets, these lncRNAs may play more important roles in cellular functions than those in nucleated cells. Of course, the roles of these individual platelet lncRNAs in platelet functions, biological processes, and disease development should be studied in the future. Nevertheless, as platelets are involved in so many biological processes and human diseases, platelet lncRNA may represent a novel research field.

## Figures and Tables

**Table 1:** The lists of 30 most abundant lncRNAs in human platelets.

Name	Gene Name	Biotype	Position	Length (bp)
ENSG00000281344	HELLPAR	macro_lncRNA	chr12:102197585-102402596:+	205,012
ENSG00000245532	NEAT1	lincRNA	chr11:65422774-65445540:+	22,679
ENSG00000251562	MALAT1	lincRNA	chr11:65497762-65506516:+	8708
ENSG00000253352	TUG1	antisense	chr22:30970677-30979395:+	5673
ENSG00000260032	LINC00657	lincRNA	chr20:36045622-36050960:−	5339
ENSG00000261026	CTD-3247F14.2	sense_overlapping	chr8:22679013-22684009:−	4997
ENSG00000215458	AATBC	antisense	chr21:43805758-43812567:−	4598
ENSG00000225733	FGD5-AS1	antisense	chr3:14920347-14948424:−	3792
ENSG00000271614	LINC00936	lincRNA	chr12:89708959-89712590:+	3632
ENSG00000231721	LINC-PINT	antisense	chr7:130941760-131110176:−	3505
ENSG00000231607	DLEU2	antisense	chr13:49982552-50125720:−	3068
ENSG00000251022	THAP9-AS1	antisense	chr4:82893009-82900960:−	2770
ENSG00000227165	WDR11-AS1	antisense	chr10:120761812-120851345:−	2500
ENSG00000270055	CTD-3092A11.2	sense_intronic	chr15:30487963-30490313:+	2351
ENSG00000265148	BZRAP1-AS1	antisense	chr17:58325450-58415766:+	2337
ENSG00000250334	LINC00989	lincRNA	chr4:79492416-79576460:+	2080
ENSG00000254614	AP003068.23	antisense	chr11:65177606-65181834:−	1662
ENSG00000234883	MIR155HG	lincRNA	chr21:25561909-25575168:+	1600
ENSG00000236304	AP001189.4	antisense	chr11:76657056-76663866:+	1426
ENSG00000229124	VIM-AS1	antisense	chr10:17214239-17229985:−	1351
ENSG00000272053	RP11-367G6.3	lincRNA	chr6:25014952-25042170:−	1030
ENSG00000253535	RP11-624C23.1	antisense	chr8:24295814-24912073:−	741
ENSG00000263934	SNORD3A	snoRNA	chr17:19188016-19188714:+	699
ENSG00000237781	RP11-54A4.2	antisense	chr1:150548562-150557724:−	665
ENSG00000262202	RP11-160E2.6	lincRNA	chr17:19112000-19112636:−	637
ENSG00000259330	INAFM2	antisense	chr15:40325216-40326715:+	594
ENSG00000254281	KB-1507C5.4	lincRNA	chr8:102978785-103000184:+	563
ENSG00000238201	AC114752.3	lincRNA	chr2:64338067-64341647:−	404
ENSG00000276216	CH17-373J23.1	lincRNA	chr1:145281116-145281462:+	347
ENSG00000273338	RP11-386I14.4	antisense	chr1:78004346-78004554:−	209

## Data Availability

RNA-seq data reported in this study are accessible through the GEO database with the GEO series accession number GSE97348. All data needed to evaluate the conclusions in the paper are present in the paper and/or the Supplementary Materials.

## Supplementary Materials

The following supporting information can be downloaded at: https://www.mdpi.com/article/10.3390/cells11142256/s1, Table S1: The lists of all differentially expressed known lncRNAs; Table S2: The lists of all differentially expressed novel lncRNAs; Table S3: The primers used in the qRT-PCR.
