# Supplementary material for: Large-Scale Profiling on lncRNAs in Human Platelets: Correlation with Platelet Reactivity
Source: Cells. 2022 Jul 21;11(14):2256. doi: 10.3390/cells11142256 (PMC9319970; doi:10.3390/cells11142256)
Supplement: Supplementary file 1 [file cells-11-02256-s001.zip › cells-1747550-supplementary.pdf]

Supplementary Materials

Table S1. The lists of all differentially expressed known lncRNAs (Hypo vs Hper). (ENSG00000258689, ENSG00000277463, ENSG00000251129 and ENSG00000226708 were highlighted and secleted for verification by qRT-PCR.)

LncRNA	length	Position	FoldChange	pval	Up/Down
ENSG00000273373	2850	chr1:110370154-110373003:+	0.4043873	0.0004814	down
ENSG00000228775	2262	chr7:141704338-141738346:-	0.3057867	0.0098213	down
ENSG00000215196	2941	chr5:17130028-17217047:-	0.1572617	0.0026069	down
ENSG00000230266	784	chr3:195147871-195152790:+	0.1501135	0.0001312	down
ENSG00000229951	3001	chr2:28384409-28394672:-	0.3502648	0.0089311	down
ENSG00000273382	2888	chr1:109087971-109090858:-	0.3387176	0.0036281	down
ENSG00000258731	1076	chr14:53153354-53157528:+	0.206406	0.0000324	down
ENSG00000235802	350	chrX:153969325-153970087:+	0.2871736	0.0024818	down
ENSG00000247728	970	chr15:30616998-30625773:-	0.4532838	0.0089271	down
ENSG00000271971	736	chr8:93715378-93716113:-	0.183472	0.0079206	down
ENSG00000255468	762	chr11:66347950-66364804:+	0.1000757	0.003018	down
ENSG00000248828	832	chr4:143953635-144126359:+	0.2840857	0.0004102	down
ENSG00000261801	2358	chr15:73908071-73928248:-	0.1223147	0.0100279	down
ENSG00000267265	786	chr19:55006193-55048086:+	0.4877105	0.0002297	down
ENSG00000163915	1924	chr3:185712528-185729787:+	0.0393154	0.0001959	down
ENSG00000255733	1791	chr12:67989445-68234686:+	0.3551071	0.0021144	down
ENSG00000241490	3023	chr3:114214313-114236204:+	0.2896927	0.0001088	down
ENSG00000249593	1067	chr5:139012647-139051203:+	0.2001513	0.0004903	down
ENSG00000229931	635	chr6:16761138-16762652:+	0.300227	0.0039078	down
ENSG00000254760	492	chr19:51414298-51414965:+	0.068802	0.0074049	down
ENSG00000234676	405	chr9:26955780-26956295:-	0.183472	0.0079206	down
ENSG00000230322	430	chr10:11168922-11171376:-	0.137604	0.0008452	down
ENSG00000277089	1115	chr17:36072866-36090134:+	0.206406	0.0032979	down
ENSG00000230257	1072	chr7:102973522-102988856:+	0.3145235	0.006109	down
ENSG00000249635	3526	chr4:106003317-106022478:-	0.0366944	0.0001089	down
ENSG00000274092	715	chr16:27313387-27314101:-	0.4892587	0.0056012	down
ENSG00000272181	418	chr3:64019508-64019925:+	0.3387176	0.0036281	down
ENSG00000248774	521	chr4:173322206-173329694:-	0.1100832	0.0000863	down
ENSG00000235843	485	chr10:26717635-26718778:+	0.308233	0.0024573	down
ENSG00000245105	2192	chr12:9065177-9068060:+	0.4631087	1.28E-07	down
ENSG00000248544	1932	chr5:157375741-157384950:-	0.1467776	0.0014867	down
ENSG00000246308	2335	chr11:10809297-10822931:+	0.183472	0.0079206	down
ENSG00000272502	1141	chr8:120812219-120813359:+	0.1000757	0.0000274	down
ENSG00000260625	655	chr16:31508471-31509256:+	0.1000757	0.003018	down
ENSG00000259985	1169	chr18:31685655-31686823:+	0.2358926	0.0092809	down
ENSG00000254418	240	chr11:14262846-14273691:-	0.1693588	0.0045542	down
ENSG00000263065	550	chr16:15741151-15741791:+	0.275208	0.0098245	down
ENSG00000236200	833	chr1:43699765-43708138:-	0.0846794	0.0009105	down
ENSG00000272195	1739	chr1:244969350-244971088:-	0.4209064	0.0049967	down
ENSG00000273002	2157	chr1:155978799-155982986:+	0.4493192	0.0015404	down
ENSG00000271857	927	chr6:45421079-45422005:-	0.4253215	0.0015603	down
ENSG00000232855	6103	chr21:28439346-28674848:-	0.3387176	0.0036281	down
ENSG00000234689	215	chr13:47905329-47907357:-	0.2896927	0.0062074	down
ENSG00000280303	2003	chr8:140636281-140638283:+	0.4403329	0.0063268	down

ENSG00000261079	2365	chr16:74367462-74369826:+	0.388529	0.0026458	down
ENSG00000278607	555	chr18:75070197-75071091:-	0.4717852	0.0026097	down
ENSG00000281852	4113	chrX:71697196-71706455:+	0.0500378	4.45E-06	down
ENSG00000223511	445	chrX:1732584-1755985:+	0.275208	0.0098245	down
ENSG00000271737	416	chr5:134005147-134005562:+	0.068802	0.0074049	down
ENSG00000230978	693	chr21:34723807-34737181:-	0.2446294	0.0037097	down
ENSG00000261584	1724	chr6:26686241-26687964:+	0.0524206	7.98E-06	down
ENSG00000249069	735	chr5:54320944-54415125:+	0.0550416	0.0021565	down
ENSG00000272693	1962	chr7:65647010-65770810:-	0.4165311	0.0031155	down
ENSG00000248243	713	chr3:130089433-130094304:-	0.1223147	0.0100279	down
ENSG00000236990	2287	chr10:4051726-4089013:+	0.4003026	0.0038576	down
ENSG00000269826	901	chr16:86347387-86349611:+	0.0611573	0.0000466	down
ENSG00000227674	1878	chr13:63986371-64076011:-	0.067595	4.01E-24	down
ENSG00000248677	3364	chr5:6686325-6707711:-	0.183472	0.0079206	down
ENSG00000245164	5330	chr8:125922308-125951249:-	0.475864	2.08E-15	down
ENSG00000278740	619	chr17:68188547-68189165:+	0.3057867	0.0098213	down
ENSG00000282828	673	chr2:49563398-49595122:+	0.4433907	0.0001003	down
ENSG00000250548	5103	chr14:61556313-61570653:-	0.3011711	2.58E-10	down
ENSG00000229656	1048	chr10:32958845-33082102:+	0.4088805	0.0034736	down
ENSG00000231971	1360	chr6:134428240-134520585:-	0.1223147	0.0002711	down
ENSG00000278376	1884	chr11:118791254-118793137:+	0.4532838	0.0089271	down
ENSG00000273344	2256	chr7:155003448-155005703:+	0.4281014	0.004454	down
ENSG00000237721	509	chr21:39006648-39011329:-	0.068802	0.0074049	down
ENSG00000282961	12727	chr8:127079874-127092600:+	0.3962996	0.0112037	down
ENSG00000254872	3139	chr11:1049880-1055749:+	0.300227	0.0004093	down
**ENSG00000226708**	**269**	**chr2:123443266-123444445:-**	**0.0550416**	**0.0021565**	**down**
ENSG00000254295	489	chr5:172954907-172957162:+	0.3387176	0.0003673	down
ENSG00000198547	5076	chr20:32631652-32673941:-	0.4077156	0.0100789	down
ENSG00000249806	512	chr4:142566019-142660950:+	0.068802	0.0074049	down
ENSG00000257151	4618	chr15:25031873-25036490:+	0.4003026	0.0008481	down
ENSG00000198685	2697	chr3:128572000-128576086:-	0.2358926	0.0092809	down
ENSG00000254615	1318	chr8:106270144-106272899:+	0.275208	0.003893	down
ENSG00000213279	2379	chr22:49572264-49575426:-	0.4403329	0.0001986	down
ENSG00000267731	2919	chr17:68189884-68192802:+	0.3490443	0.000346	down
ENSG00000205663	2194	chrX:3891438-3920746:-	0.2635795	2.69E-08	down
ENSG00000242659	303	chr3:113746872-113747408:-	0.068802	0.0074049	down
ENSG00000269887	715	chr1:164680085-164680799:+	0.4953745	0.0100409	down
ENSG00000274275	325	chr18:32031035-32031359:+	0.275208	0.0098245	down
ENSG00000267749	2016	chr19:2641838-2643853:-	0.1000757	0.003018	down
ENSG00000261560	1495	chr16:11881075-11882569:-	0.1000757	0.003018	down
ENSG00000261288	572	chr16:2554975-2556105:+	0.4953745	0.0100409	down
ENSG00000231125	794	chr21:29058073-29060095:-	0.091736	0.0016569	down
ENSG00000269911	1490	chrX:72777608-72779097:+	0.1100832	0.0055007	down
ENSG00000253838	102	chr8:39914229-39915721:+	0.2555503	5.33E-07	down
ENSG00000260190	820	chr9:136937169-136937988:-	0.3669441	0.003157	down
ENSG00000236310	386	chr7:41667168-41705335:-	0.068802	0.0074049	down
ENSG00000239801	364	chr3:57628810-57654918:+	12.109154	0.0013114	up
ENSG00000225981	498	chr7:1459937-1464008:+	2.6909231	0.0086101	up
ENSG00000233030	3543	chr1:149785659-149793020:-	3.1452347	0.0052912	up
ENSG00000235527	2117	chr1:113924000-113929492:-	2.0444026	0.0067972	up

ENSG00000246228	1420	chr8:127289817-127482139:-	2.9355524	0.000744	up
ENSG00000225542	705	chr3:21542816-21579959:+	8.2562412	0.0004733	up
ENSG00000257696	418	chr12:100143058-100144671:+	7.155409	0.0016669	up
ENSG00000254733	411	chr11:86431590-86622867:+	17.613315	0.0041007	up
ENSG00000240005	482	chr4:26859806-26860599:-	9.9074895	0.0049493	up
ENSG00000274751	3867	chr16:1305547-1309413:-	8.8066573	0.0096361	up
ENSG00000224424	3447	chr3:48847572-48851981:+	4.6785367	0.0017191	up
ENSG00000230910	2365	chr6:68629962-68635027:-	2.7520804	5.63E-05	up
ENSG00000267296	2198	chr19:33302857-33305054:+	2.2567059	0.0020595	up
ENSG00000255306	739	chr11:68024809-68030461:-	8.8066573	0.0002514	up
ENSG00000257599	963	chr12:29389294-29487488:+	22.016643	1.92E-34	up
ENSG00000225721	580	chr1:44759037-44775810:-	8.8066573	0.0096361	up
ENSG00000259436	1955	chr19:42152569-42157523:+	2.0365395	0.0002058	up
ENSG00000255446	510	chr11:62421845-62426724:-	2.3589261	0.0059818	up
ENSG00000251139	628	chr4:184813619-184821300:+	2.0548867	0.001105	up
ENSG00000261758	3151	chr3:136752630-136755780:+	4.7702727	0.0057369	up
ENSG00000272158	443	chr14:65003325-65003767:-	5.5041608	0.010717	up
ENSG00000272509	462	chr8:94884609-94885070:+	6.0545769	0.0057913	up
ENSG00000233070	500	chrY:2966844-3002626:-	11.008322	0.0025455	up
ENSG00000271797	944	chr5:115262505-115263448:+	28.621636	0.0001511	up
ENSG00000273261	699	chr3:182921240-182921938:-	22.016643	0.0010714	up
ENSG00000274877	3231	chr2:113237595-113240825:+	3.9140699	6.97E-05	up
ENSG00000253796	486	chr8:108895029-109063417:-	3.3024965	0.0064331	up
ENSG00000269890	3955	chr1:228270443-228274397:-	2.3851363	0.0097444	up
ENSG00000247498	1746	chr12:12927726-12984645:+	3.61702	0.0011947	up
ENSG00000271474	3329	chr4:95549129-95552457:+	5.5041608	0.010717	up
ENSG00000282386	710	chr1:153964361-153965070:+	4.4033286	0.0101991	up
ENSG00000261351	2168	chr15:66488658-66492109:-	2.0721547	0.0127911	up
ENSG00000274227	627	chr12:112018804-112019430:-	2.6419972	0.0068202	up
ENSG00000228201	389	chr16:648473-649200:-	3.3024965	0.0128668	up
ENSG00000214772	657	chr12:14665655-14757963:+	5.5041608	0.010717	up
ENSG00000233672	1707	chr13:50862172-50910764:-	4.1831622	0.0015722	up
ENSG00000281128	873	chr9:33677268-33688011:+	5.5041608	0.010717	up
ENSG00000226609	420	chr9:112748902-112750456:-	9.9074895	0.0049493	up
ENSG00000224649	327	chr21:29182027-29187795:-	2.2802952	0.008847	up
ENSG00000227407	529	chr19:54430654-54434698:-	2.3851363	0.0097444	up
ENSG00000276136	848	chr12:32000375-32001222:+	2.3667891	0.0009637	up
ENSG00000271324	502	chr3:33793644-33794145:-	7.155409	0.0016669	up
ENSG00000232850	2100	chr9:128128529-128130628:+	11.008322	0.0025455	up
ENSG00000198468	2525	chr1:212852108-212858088:-	4.4033286	8.60E-06	up
ENSG00000257900	548	chr14:45377268-45389286:+	2.3484419	0.0046937	up
ENSG00000228106	768	chr1:222815022-222837384:+	2.1015887	0.0037663	up
ENSG00000212743	2447	chr10:6350316-6352762:+	2.2934003	0.0005474	up
ENSG00000228437	1148	chr1:221966341-221984964:+	4.7702727	0.0057369	up
ENSG00000259005	589	chr14:74474007-74474864:-	2.3589261	0.0059818	up
ENSG00000227925	423	chr1:221827666-221840666:-	7.7058251	0.0008894	up
ENSG00000251615	3249	chr4:8355090-8358338:-	2.0793496	0.0099914	up
ENSG00000272360	462	chr3:58490830-58491291:-	2.8896844	0.0068726	up
ENSG00000255363	559	chr11:76628096-76630363:-	2.0746452	0.0020369	up
ENSG00000250072	3091	chr5:149063317-149109787:+	4.5868007	0.0001623	up

ENSG00000203729	740	chr1:182407621-182414815:+	17.613315	0.0041007	up
ENSG00000256433	1803	chr12:6393905-6396148:+	2.2016643	0.0079045	up
ENSG00000237357	2579	chr9:42566679-42569353:-	9.9074895	0.0049493	up
ENSG00000271789	1385	chr6:111297126-111298510:+	2.2016643	0.0014497	up
ENSG00000275759	590	chr12:118428281-118428870:+	13.209986	0.000677	up
ENSG00000253139	555	chr8:56536758-56540497:-	5.1372168	0.0032026	up
**ENSG00000258689**	**602**	**chr14:70698698-70712153:+**	**17.613315**	**0.0041007**	**up**
ENSG00000248489	478	chr5:98929171-98995013:+	3.8529126	0.0090385	up
ENSG00000254837	2493	chr11:74493366-74498533:+	2.2016643	0.0129707	up
ENSG00000258216	547	chr12:89882665-89989289:+	8.8066573	0.0096361	up
ENSG00000255621	2403	chr12:13000451-13040679:+	11.008322	0.0025455	up
ENSG00000236611	346	chr22:23326616-23328493:+	6.604993	0.0031134	up
ENSG00000267940	1353	chr11:6201901-6203253:-	2.5254385	0.0008811	up
ENSG00000226032	331	chr6:159064728-159065273:-	4.4033286	0.0101991	up
ENSG00000268518	626	chr19:50486810-50487638:-	2.0181923	0.0015433	up
**ENSG00000277463**	**423**	**chr17:62626031-62626453:+**	**17.613315**	**0.0041007**	**up**
ENSG00000269051	545	chr19:53197111-53211015:+	2.715386	0.0006027	up
ENSG00000226816	2033	chr7:23206013-23208045:+	2.4375569	0.004011	up
ENSG00000248636	695	chr12:119387987-119668079:-	2.0793496	0.0016044	up
ENSG00000214145	2880	chr3:194296465-194312803:-	6.0545769	0.0057913	up
ENSG00000273211	479	chr3:48985485-48985963:-	17.613315	0.0041007	up
ENSG00000272203	753	chr5:132817248-132818000:-	9.9074895	0.0049493	up
ENSG00000232324	555	chr19:53864763-53866140:+	8.8066573	0.0096361	up
ENSG00000257747	1372	chr12:81953719-81993133:+	2.7520804	0.0108525	up
ENSG00000234902	823	chr2:207186717-207236066:-	2.6909231	0.0086101	up
**ENSG00000251129**	**734**	**chr4:31997397-32155406:+**	**4.1831622**	**0.0015722**	**up**
ENSG00000254952	385	chr11:60159687-60160822:-	2.2016643	0.0129707	up
ENSG00000270000	431	chr14:74471930-74472360:-	4.4033286	2.73E-05	up
ENSG00000237803	1923	chr2:37826247-37876274:-	2.1303141	5.43E-11	up
ENSG00000250057	394	chr4:83233512-83247213:-	2.0580775	0.0041866	up
ENSG00000256955	770	chr12:131857420-131864538:+	3.4859685	0.0038902	up
ENSG00000166770	1521	chr19:56477250-56500666:+	2.6419972	1.89E-05	up
ENSG00000224050	590	chr22:32273420-32277186:+	5.5041608	0.010717	up
ENSG00000266934	716	chr17:61382785-61384680:+	3.5226629	0.0077113	up
ENSG00000276248	3362	chr13:113527260-113530621:+	2.2016643	0.0101178	up
ENSG00000270077	554	chr8:97144170-97144723:+	2.8896844	0.0001321	up
ENSG00000267285	574	chr12:1385833-1386987:+	2.987973	0.0085391	up
ENSG00000259735	497	chr15:59348648-59359889:-	2.5018913	0.0082377	up
ENSG00000224356	2367	chr13:100535741-100587146:-	2.2016643	0.0129707	up
ENSG00000266371	2780	chr17:31133182-31138518:+	2.2567059	0.0020595	up
ENSG00000260293	5616	chr16:2476558-2482173:+	15.41165	0.0081245	up
ENSG00000259999	427	chr16:75321890-75325048:-	5.2289528	0.000549	up
ENSG00000224992	1019	chr9:132768965-132770212:-	22.016643	0.0010714	up
ENSG00000277558	583	chr20:34476205-34476787:+	2.987973	0.0085391	up
ENSG00000229832	496	chr1:212357418-212358353:+	19.814979	0.0020877	up
ENSG00000229521	428	chr13:77080511-77081190:-	2.2016643	0.0061839	up
ENSG00000276107	733	chr15:39586561-39587293:+	2.0677793	2.26E-07	up
ENSG00000215533	989	chr21:29193480-29288205:+	2.0548867	0.001105	up
ENSG00000251405	1992	chr5:157362615-157460078:-	4.4033286	0.0101991	up

Table S2. The lists of all differentially expressed novel lncRNAs (Hypo vs Hper)(lnc_417 and lnc_1129 were highlighted and secleted for verification by qRT-PCR.)

Gene	Hypo_normalize	Hyper_normalize	FoldChange	Log2FoldChange	pval	Up/Down
lnc_330	290.5803	11080.29	0.026225	-5.25291	0	down
lnc_196	95.03254	2985.677	0.031829	-4.97349	0	down
lnc_793	619.5391	4182.571	0.148124	-2.75512	0	down
lnc_84	16.44794	2290.494	0.007181	-7.12161	1.53E-299	down
lnc_889	169.962	1059.169	0.160467	-2.63965	2.17E-91	down
lnc_305	427.6464	1365.771	0.313117	-1.67523	7.69E-67	down
lnc_270	40.20607	552.5387	0.072766	-3.78059	2.31E-66	down
lnc_710	246.7191	970.6318	0.254184	-1.97605	9.80E-60	down
lnc_57	511.7137	1231.325	0.41558	-1.2668	2.00E-40	down
lnc_9	98.68764	455.8035	0.216514	-2.20747	4.20E-33	down
lnc_596	681.6757	1383.806	0.492609	-1.02148	1.62E-32	down
lnc_612	537.2994	1118.194	0.480506	-1.05737	4.83E-28	down
lnc_677	12.79284	211.5059	0.060485	-4.04729	1.40E-27	down
**lnc_417**	**0.913774**	**196.7497**	**0.004644**	**-7.75031**	**3.69E-26**	**down**
lnc_896	12.79284	186.9122	0.068443	-3.86895	6.35E-24	down
lnc_187	354.5445	796.8363	0.44494	-1.16832	9.74E-24	down
lnc_841	63.96421	298.4037	0.214355	-2.22193	2.10E-22	down
lnc_814	0.913774	141.0039	0.00648	-7.26968	6.79E-20	down
lnc_608	274.1323	568.9345	0.481835	-1.05339	5.92E-15	down
lnc_749	32.89588	167.2372	0.196702	-2.34592	4.99E-14	down
lnc_1084	16.44794	118.0498	0.13933	-2.84342	1.97E-12	down
lnc_1025	0.913774	70.50197	0.012961	-6.26968	3.54E-11	down
lnc_938	16.44794	103.2936	0.159235	-2.65077	2.23E-10	down
lnc_227	102.3427	252.4954	0.405325	-1.30285	7.34E-10	down
lnc_1232	91.37744	229.5413	0.398087	-1.32884	2.49E-09	down
lnc_607	74.9295	201.6684	0.371548	-1.42838	3.54E-09	down
lnc_1154	7.310195	70.50197	0.103688	-3.26968	6.32E-09	down
lnc_483	29.24078	116.4102	0.251187	-1.99316	1.38E-08	down
lnc_287	21.93059	91.81652	0.238852	-2.06581	2.48E-07	down
lnc_649	29.24078	101.654	0.28765	-1.79761	7.48E-07	down
lnc_626	47.51627	132.806	0.357787	-1.48283	8.34E-07	down
lnc_648	23.75813	90.17694	0.263461	-1.92434	1.06E-06	down
lnc_402	47.51627	131.1665	0.362259	-1.46491	1.22E-06	down
lnc_364	74.9295	173.7956	0.431136	-1.21379	1.34E-06	down
lnc_1140	20.10304	77.0603	0.260874	-1.93857	5.78E-06	down
lnc_522	20.10304	77.0603	0.260874	-1.93857	5.78E-06	down
lnc_542	21.93059	78.69988	0.278661	-1.84342	9.44E-06	down
lnc_261	87.72234	181.9935	0.482008	-1.05287	1.02E-05	down
lnc_1261	38.37853	101.654	0.377541	-1.4053	3.46E-05	down
lnc_477	16.44794	60.66449	0.27113	-1.88295	7.97E-05	down
lnc_906	52.99892	118.0498	0.448954	-1.15536	0.00012769	down
lnc_1273	9.137744	42.6291	0.214355	-2.22193	0.00023322	down
lnc_202	10.96529	45.90826	0.238852	-2.06581	0.00026431	down
lnc_633	3.655098	29.51245	0.123849	-3.01334	0.00029415	down
lnc_770	9.137744	40.98952	0.222929	-2.16535	0.00038332	down

lnc_296	21.93059	63.94365	0.342967	-1.54386	0.00042808	down
lnc_603	18.27549	57.38533	0.31847	-1.65077	0.00046143	down
lnc_209	45.68872	100.0144	0.456821	-1.1303	0.00053099	down
lnc_192	9.137744	39.34994	0.232217	-2.10645	0.00062703	down
lnc_1090	7.310195	34.4312	0.212313	-2.23573	0.00090053	down
lnc_848	18.27549	54.10617	0.337771	-1.56588	0.00106189	down
lnc_883	40.20607	86.89778	0.462682	-1.11191	0.00143744	down
lnc_715	49.34382	100.0144	0.493367	-1.01927	0.00144764	down
lnc_1148	14.62039	45.90826	0.31847	-1.65077	0.00173351	down
lnc_343	10.96529	39.34994	0.278661	-1.84342	0.00173505	down
lnc_1262	7.310195	31.15204	0.234662	-2.09134	0.00245574	down
lnc_332	10.96529	37.71036	0.290777	-1.78202	0.00273707	down
lnc_801	23.75813	59.02491	0.40251	-1.3129	0.00274226	down
lnc_480	34.72343	73.78114	0.470627	-1.08734	0.00392628	down
lnc_1008	25.58568	59.02491	0.433473	-1.20599	0.00506644	down
lnc_750	36.55098	73.78114	0.495397	-1.01334	0.00648022	down
lnc_679	0.913774	13.11665	0.069665	-3.84342	0.00767553	down
lnc_313	3.655098	19.67497	0.185774	-2.42838	0.00838059	down
lnc_647	27.41323	59.02491	0.464435	-1.10645	0.00889166	down
lnc_517	20.10304	47.54784	0.422796	-1.24197	0.01003827	down
lnc_1000	0.913774	11.47707	0.079617	-3.65077	0.01431555	down
lnc_523	23.75813	50.827	0.467431	-1.09717	0.01589868	down
lnc_259	9.137744	27.87287	0.327836	-1.60895	0.016564	down
lnc_954	7.310195	24.59371	0.297238	-1.75031	0.01687685	down
lnc_1207	21.93059	47.54784	0.461232	-1.11644	0.01802096	down
lnc_965	20.10304	44.26868	0.454114	-1.13887	0.02039499	down
lnc_1147	61166.23	9181.652	6.661789	2.73591	0	up
lnc_94	5478.991	6.558323	835.4257	9.706368	0	up
lnc_471	3200.038	78.69988	40.66128	5.345584	0	up
lnc_1285	5674.539	1733.037	3.274333	1.711201	2.22E-280	up
lnc_586	1478.487	18.03539	81.977	6.357147	1.68E-201	up
lnc_290	1780.033	186.9122	9.523362	3.251471	1.97E-186	up
lnc_880	1160.494	42.6291	27.22304	4.766756	8.35E-155	up
lnc_1185	1019.772	14.75623	69.10793	6.110779	1.73E-140	up
lnc_1003	1118.46	72.14155	15.50368	3.954539	2.54E-136	up
lnc_1100	1560.727	339.3932	4.598579	2.201188	1.13E-108	up
lnc_655	515.3688	6.558323	78.5824	6.296134	1.23E-71	up
lnc_4	1668.552	631.2386	2.643299	1.402339	5.06E-63	up
lnc_756	369.1649	6.558323	56.28952	5.814794	3.20E-52	up
lnc_153	509.8861	73.78114	6.910793	2.788851	1.60E-47	up
lnc_1107	498.9208	77.0603	6.474421	2.694751	5.91E-45	up
lnc_949	749.295	200.0289	3.745935	1.905326	2.10E-44	up
lnc_1007	1337.766	559.0971	2.392725	1.258655	2.37E-43	up
lnc_997	643.2972	162.3185	3.963179	1.986658	1.87E-40	up
lnc_467	453.2321	104.9332	4.319245	2.110779	3.19E-31	up
lnc_742	855.2929	345.9515	2.472291	1.305848	6.83E-30	up
lnc_252	266.8221	45.90826	5.812072	2.539053	2.31E-23	up
lnc_1149	179.0998	24.59371	7.282341	2.864402	3.33E-18	up
lnc_531	168.1345	26.23329	6.409203	2.680145	3.86E-16	up

lnc_657	126.1009	9.837485	12.81841	3.680145	3.88E-16	up
lnc_864	416.6811	191.831	2.172127	1.119108	2.28E-12	up
lnc_1051	215.6508	65.58323	3.2882	1.717298	2.91E-12	up
lnc_423	138.8937	31.15204	4.458576	2.156583	6.99E-11	up
**lnc_1129**	**58.48156**	**0.81979**	**71.33721**	**6.156583**	**1.55E-09**	**up**
lnc_75	129.756	32.79162	3.956986	1.984402	2.30E-09	up
lnc_458	52.99892	0.81979	64.64935	6.014564	8.57E-09	up
lnc_1101	51.17137	0.81979	62.42006	5.963938	1.53E-08	up
lnc_347	60.30911	6.558323	9.195813	3.200977	1.03E-07	up
lnc_1272	191.8926	78.69988	2.438284	1.285866	1.07E-07	up
lnc_1132	42.03362	0.81979	51.27362	5.680145	2.89E-07	up
lnc_519	51.17137	4.918742	10.40334	3.378975	5.23E-07	up
lnc_79	38.37853	0.81979	46.81505	5.5489	9.62E-07	up
lnc_858	206.513	96.73527	2.134827	1.094119	1.24E-06	up
lnc_766	67.61931	13.11665	5.155228	2.366036	1.48E-06	up
lnc_663	49.34382	6.558323	7.523847	2.91147	4.15E-06	up
lnc_870	63.96421	13.11665	4.876567	2.285866	4.51E-06	up
lnc_157	51.17137	8.197904	6.242006	2.64201	8.29E-06	up
lnc_1036	113.308	42.6291	2.657997	1.41034	1.17E-05	up
lnc_1215	109.6529	40.98952	2.675146	1.419617	1.47E-05	up
lnc_69	65.79176	16.39581	4.012718	2.00458	1.84E-05	up
lnc_571	29.24078	0.81979	35.66861	5.156583	2.10E-05	up
lnc_58	137.0662	60.66449	2.259413	1.175948	2.83E-05	up
lnc_892	29.24078	1.639581	17.8343	4.156583	4.55E-05	up
lnc_599	124.2733	54.10617	2.296842	1.199652	5.12E-05	up
lnc_132	62.13666	18.03539	3.445263	1.784614	0.00011838	up
lnc_286	45.68872	9.837485	4.64435	2.215477	0.00014296	up
lnc_1274	40.20607	8.197904	4.904433	2.294086	0.00026832	up
lnc_1188	113.308	52.46659	2.159623	1.110779	0.00027658	up
lnc_1208	67.61931	24.59371	2.749455	1.459146	0.00051305	up
lnc_600	40.20607	9.837485	4.087028	2.031052	0.00073348	up
lnc_366	34.72343	8.197904	4.235647	2.082582	0.00142721	up
lnc_918	34.72343	9.837485	3.529706	1.819548	0.00354274	up
lnc_555	58.48156	24.59371	2.377907	1.249692	0.0041159	up
lnc_849	58.48156	24.59371	2.377907	1.249692	0.0041159	up
lnc_567	16.44794	1.639581	10.0318	3.326508	0.00471254	up
lnc_936	60.30911	26.23329	2.298953	1.200977	0.004746	up
lnc_857	23.75813	4.918742	4.830124	2.27206	0.00535349	up
lnc_149	20.10304	3.279162	6.130542	2.616015	0.00545382	up
lnc_406	40.20607	14.75623	2.724685	1.44609	0.00779771	up
lnc_581	34.72343	11.47707	3.025462	1.597156	0.00780513	up
lnc_327	25.58568	6.558323	3.901254	1.963938	0.00840967	up
lnc_234	25.58568	6.558323	3.901254	1.963938	0.00840967	up
lnc_946	36.55098	13.11665	2.78661	1.478511	0.0099022	up
lnc_879	51.17137	22.95413	2.229288	1.156583	0.01159389	up
lnc_796	38.37853	14.75623	2.600836	1.378975	0.01214571	up
lnc_1192	40.20607	16.39581	2.452217	1.294086	0.01450201	up
lnc_1281	47.51627	21.31455	2.229288	1.156583	0.01499729	up
lnc_280	29.24078	9.837485	2.972384	1.57162	0.01582377	up

lnc_760	20.10304	4.918742	4.087028	2.031052	0.01695367	up
lnc_1181	12.79284	1.639581	7.802508	2.963938	0.01804505	up

Table S3. The primers used in the qRT-PCR.

lncRNA name	Genetic location	lengths(nt)	Quantitative PCR primer pairs
ENSG00000258689	chr14:70698698-70712153:+	602	Forward:5'-CAAGCCCACTGTGCTGATTT-3'<br>Reverse: 5'-GAGCCCAGGAGTTTGAGACC-3'
ENSG00000277463	chr17:62626031-62626453:+	423	Forward:5'-GGAGCCACAGCCACCAGAAG-3'<br>Reverse: 5'-TGGAAAGGGTCCCATTAGCC-3'
ENSG00000251129	chr4:31997397-32155406:+	734	Forward:5'-GGGCAGACTACCCACCTAAC-3'<br>Reverse: 5'-AGGAGGCTGAGATGGATGAA-3'
ENSG00000226708	chr2:123443266-123444445:-	269	Forward:5'-GCTGCTTCTTCCAACCTCAG-3'<br>Reverse: 5'-TTCTACACTTCCATTTCCATTA-3'
lnc1129	chr20:45435272-45448325:-	1794	Forward:5'-ATGACCATCTTCTTATTGACCCTC-3'<br>Reverse: 5'-CCTGGCTCACTCCTGCTTCT-3'
lnc417	chr7:19018-31194:-	600	Forward:5'-AAACTGTGAGCAAGAGCGTCTG-3'<br>Reverse: 5'-GCCATCAATCATTTCATTAGCGTA-3'
